# Supplementary material for: Trends in hepatitis C virus coinfection and its cascade of care among adults living with HIV in Asia between 2010 and 2020
Source: PLoS One. 2023 Jun 28;18(6):e0287909. doi: 10.1371/journal.pone.0287909 (PMC10306215; doi:10.1371/journal.pone.0287909)
Supplement: Attachment — Submitted filename: Response to reviewers.doc [file pone.0287909.s001.doc]

20 April 2023

Chen-Hua Liu

Academic Editor

PLOS ONE

Dear Dr. Liu and Reviewers,

On behalf of myself and my co-authors, thank you for the opportunity to revise and resubmit our paper, “**Trends in hepatitis C virus coinfection and its cascade of care among adults living with HIV in Asia between 2010 and 2020,**” to PLOS ONE. Please see all your comments addressed point-by-point below, starting from the next page of this letter.

We appreciate your review, comments, and further consideration of our revised manuscript.

Regards,

Jeremy L. Ross, MBBS, MSc – Corresponding author

Director of Research

TREAT Asia/amfAR – The Foundation for AIDS Research

Bangkok, Thailand

[jeremy.ross@treatasia.org](mailto:jeremy.ross@treatasia.org)

**Academic editor comments and responses**

**Please ensure that your manuscript meets PLOS ONE's style requirements, including those for file naming.** Response: We have reviewed and confirm that our manuscript meets PLOS ONE's style requirements, including those for file naming.

**Thank you for stating the following in the Competing Interests section: "AHS has received grants to her institution from ViiV Healthcare and Gilead Sciences. Other authors have declared that no competing interests exist.” Please confirm that this does not alter your adherence to all PLOS ONE policies on sharing data and materials, by including the following statement: "This does not alter our adherence to PLOS ONE policies on sharing data and materials.”** Response: We have updated our Competing Interests section to include the above statement and provided it in the revised cover letter, as requested.

**We note that you have indicated that data from this study are available upon request. PLOS only allows data to be available upon request if there are legal or ethical restrictions on sharing data publicly.** Response: We have provided the ethical or legal restrictions on sharing our de-identified data set in the revised cover letter, as requested.

**One of the noted authors is a group or consortium "TAHOD-LITE study group of IeDEA Asia‐Pacific". In addition to naming the author group, please list the individual authors and affiliations within this group in the acknowledgments section of your manuscript. Please also indicate clearly a lead author for this group along with a contact email address.** Response: The individual authors for this group and their affiliations have been provided. We indicated a lead author for this group and their contact email address in the revised manuscript, as requested.

**Please include your full ethics statement in the ‘Methods’ section of your manuscript file. In your statement, please include the full name of the IRB or ethics committee who approved or waived your study, as well as whether or not you obtained informed written or verbal consent. If consent was waived for your study, please include this information in your statement as well.** Response: We have replaced the previous ethics statement with the following full ethics statement that includes the above information, in the ‘Methods’ section of our manuscript, as requested: *“The following IRBs approved the study: Cambodia Ministry of Health National Ethics Committee for Health Research; Kowloon Central / Kowloon East Research Ethics Committee; VHS Institutional Ethics Committee; Institutional Ethics Committee Rao Nursing Home; Kerti Praja Foundation IRB; Severance Hospital Yonsei University College of Medicine IRB; The Ethical Committee for Research in Human Subject, Chiangrai Prachanukroh Hospital; Institutional Review Board Faculty of Medicine, Chulalongkorn University; Committee on Human Rights Related to Research Involving Human Subjects, Faculty of Medicine Ramathibodi Hospital; Hanoi School of Public Health IRB; The Ethical Review Board for Biomedical Research of National Hospital of Tropical Diseases; UNSW Human Research Ethics Committee; and Advarra IRB. Written informed consent was obtained at the following study sites: Chiangrai Prachanukroh Hospital; HIV-NAT, the Thai Red Cross AIDS Research Centre; and Ramathibodi Hospital. Consent at all other sites was waived due to the observational nature of the study, and because data was only collected retrospectively from available medical records.”*

**Please review your reference list to ensure that it is complete and correct.** Response: We have reviewed the reference list and confirm it is complete and correct**.**

**Reviewer #1 comments and responses**

**Overall: This study from a prospective observational cohort of the International Epidemiology Databases to Evaluate AIDS (IeDEA) Asia-Pacific (TAHOD-LITE) was aimed to assess regional HCV coinfection and cascade outcomes among adults living with HIV in care from 2010-2020 in Asia.**

Response: Thank you for your review of our manuscript and recommendations to improve it. We hope these have been adequately addressed in our responses (below) and associated edits.

**Comment 1. The proportion of people living with HIV (PLHIV) with a positive anti-HCV test was reported over three time periods that broadly correspond to very limited DAA access (the pre-DAA era), initial DAA access, and expanded DAA access in the Asia-Pacific region: 2010-2014, 2015-2017, and 2018-2020. Results of three time periods should be mentioned in Abstract.**

Response: We have added data on the positive anti-HCV test results and HCV cascade proportions from all three of the 2010-2014, 2015-2017, and 2018-2020 time periods to the abstract.

**Comment 2. Liver fibrosis, liver function reserve, renal function, HCV genotype and HCV RNA may be factors associated with chronic HCV treatment response. The proportion of liver cirrhosis, liver fibrosis grade, serum albumin, creatinine, HCV genotype and HCV RNA should be analyzed in Table 4.**

Response: Thank you for this suggestion to include these additional factors in the chronic HCV treatment response regression analysis. As suggested we did have adequate creatinine data and have added that to the analysis. However, we were either unable to include the other factors or felt it could confuse the findings. We believe that associations between liver cirrhosis and fibrosis and chronic HCV treatment response are likely already covered to an extent by our inclusion of ALT and AST levels. Unfortunately, we did not collect albumin test results from our study participants so cannot add that to the regression analysis. In addition, HCV genotype results are very limited or often not available, particularly from sites in resource-limited countries or countries that use pan-genotypic DAAs and rarely test HCV genotype. Regarding HCV RNA, we expect more HCV RNA testing to be done in later calendar years which would confound with the calendar year variable included. Therefore, instead of including it, HCV RNA has been discussed. Furthermore, inclusion of HCV RNA in our analysis will likely show that high HCV RNA is associated with a reduced hazard for achieving SVR. Table 4 and the associated text in the methods and results sections have been revised to reflect the changes that were made.

**Comment 3. Table 2, Calendar year, “2017”-2020 should be “2018”-2020.**

Response: Thank you for spotting this error, which we have now corrected.
