# Supplementary material for: Trends in hepatitis C virus coinfection and its cascade of care among adults living with HIV in Asia between 2010 and 2020
Source: PLoS One. 2023 Jun 28;18(6):e0287909. doi: 10.1371/journal.pone.0287909 (PMC10306215; doi:10.1371/journal.pone.0287909)
Supplement: Attachment — Submitted filename: Response to reviewers.doc [file pone.0287909.s002.doc]

14 June 2023

Chen-Hua Liu

Academic Editor

PLOS ONE

Dear Dr. Liu,

On behalf of myself and my co-authors, thank you for the opportunity to further revise and resubmit our paper, “**Trends in hepatitis C virus coinfection and its cascade of care among adults living with HIV in Asia between 2010 and 2020,**” to PLOS ONE. Please see all comments addressed point-by-point below, starting from the next page of this letter.

We appreciate your further consideration of our revised manuscript.

Regards,

Jeremy L. Ross, MBBS, MSc – Corresponding author

Director of Research

TREAT Asia/amfAR – The Foundation for AIDS Research

Bangkok, Thailand

[jeremy.ross@treatasia.org](mailto:jeremy.ross@treatasia.org)

**Additional editor comments and responses**

**Please make a brief discussion about the potential HCV reinfection among PLWH after achieving SVR with antiviral treatment. In Taiwan, a recent study showed that a increasing trend of HCV reinfection among HIV positive patients after the commencement of direct-acting antivirals, which may post a public health treat and should alert the physicians about the post-treatment surveillance based on public health perspectives (Liu CH, et al. Open Forum Infect Dis 2022).**

Response: Thank you for the suggestion to add to the discussion the potential for HCV reinfection and the importance of post HCV treatment surveillance among those living with HIV, and for providing an associated reference. The following text was added to the discussion section: *“Improved HCV screening and scale-up of HCV treatment are also important for reducing the risk of HCV re-infection amongst PLHIV (56). A recent study of HCV re-infection among an adult PLHIV cohort in Taiwan found that those living with HIV had higher HCV re-infection rates than the HIV-negative reference population, and that HCV reinfection incidence increased following the availability of DAAs in the country, further highlighting the importance of post HCV treatment surveillance among PLHIV (57).”* The following references were also added: *“56. Hosseini-Hooshyar S, Hajarizadeh B, Bajis S, Law M, Janjua NZ, Fierer DS, et al. Risk of hepatitis C reinfection following successful therapy among people living with HIV: a global systematic review, meta-analysis, and meta-regression. The lancet HIV. 2022;9(6):e414-e27”* and *“57. Liu CH, Sun HY, Peng CY, Hsieh SM, Yang SS, Kao WY, et al. Hepatitis C Virus Reinfection in People With HIV in Taiwan After Achieving Sustained Virologic Response With Antiviral Treatment: The RECUR Study. Open Forum Infect Dis. 2022;9(8):ofac348.”*
